# Two microbiota subtypes identified in irritable bowel syndrome with distinct responses to the low FODMAP diet

**DOI:** 10.1136/gutjnl-2021-325177

**Published:** 2021-11-22

**Authors:** Kevin Vervier, Stephen Moss, Nitin Kumar, Anne Adoum, Meg Barne, Hilary Browne, Arthur Kaser, Christopher J Kiely, B Anne Neville, Nina Powell, Tim Raine, Mark D Stares, Ana Zhu, Juan De La Revilla Negro, Trevor D Lawley, Miles Parkes

**Affiliations:** 1 Host-Microbiota Interactions Laboratory, Wellcome Sanger Institute, Hinxton, Cambridgeshire, UK; 2 Department of Gastroenterology, Addenbrookes Hospital, Cambridge, UK; 3 Division of Gastroenterology and Hepatology, Department of Medicine, University of Cambridge, Cambridge, Cambridgeshire, UK; 4 Department of Dietetics, Addenbrookes Hospital, Cambridge, UK; 5 Cambridge Institute of Therapeutic Immunology and Infectious Disease, University of Cambridge, Cambridge, Cambridgeshire, UK; 6 Department of Gastroenterology, Royal North Shore Hospital, St Leonards, New South Wales, Australia; 7 Wellcome Sanger Institute, Hinxton, Cambridgeshire, UK

**Keywords:** intestinal microbiology, diet, irritable bowel syndrome

## Abstract

**Objective:**

Reducing FODMAPs (fermentable oligosaccharides, disaccharides, monosaccharides and polyols) can be clinically beneficial in IBS but the mechanism is incompletely understood. We aimed to detect microbial signatures that might predict response to the low FODMAP diet and assess whether microbiota compositional and functional shifts could provide insights into its mode of action.

**Design:**

We used metagenomics to determine high-resolution taxonomic and functional profiles of the stool microbiota from IBS cases and household controls (n=56 pairs) on their usual diet. Clinical response and microbiota changes were studied in 41 pairs after 4 weeks on a low FODMAP diet.

**Results:**

Unsupervised analysis of baseline IBS cases pre-diet identified two distinct microbiota profiles, which we refer to as IBS^P^ (pathogenic-like) and IBS^H^ (health-like) subtypes. IBS^P^ microbiomes were enriched in Firmicutes and genes for amino acid and carbohydrate metabolism, but depleted in Bacteroidetes species. IBS^H^ microbiomes were similar to controls. On the low FODMAP diet, IBS^H^ and control microbiota were unaffected, but the IBS^P^ signature shifted towards a health-associated microbiome with an increase in Bacteroidetes (p=0.009), a decrease in Firmicutes species (p=0.004) and normalisation of primary metabolic genes. The clinical response to the low FODMAP diet was greater in IBS^P^ subjects compared with IBS^H^ (p=0.02).

**Conclusion:**

50% of IBS cases manifested a ‘pathogenic’ gut microbial signature. This shifted towards the healthy profile on the low FODMAP diet; and IBS^P^ cases showed an enhanced clinical responsiveness to the dietary therapy. The effectiveness of FODMAP reduction in IBS^P^ may result from the alterations in gut microbiota and metabolites produced. Microbiota signatures could be useful as biomarkers to guide IBS treatment; and investigating IBS^P^ species and metabolic pathways might yield insights regarding IBS pathogenic mechanisms.

**Significance of this study**

**What is already known on this subject?**

- Patients with IBS often respond to a low FODMAP (fermentable oligosaccharides, disaccharides, monosaccharides and polyols) diet.
- The gut microbiota has been implicated in IBS.
- The microbiota in patients with IBS may change with diet.

**What are the new findings?**

- We were able to stratify patients with IBS according to their gut microbiota species and metabolic gene signatures.
- We identified a distinct gut microbiota subtype with an enhanced clinical response to a low FODMAP diet compared with other subjects with IBS.

**How might it impact on clinical practice in the foreseeable future?**

- The potential development of a microbiota signature as a biomarker to manage IBS cases with a low FODMAP diet recommendation.
- If the bacteria represented in the IBS^P^ subtype are shown to play a pathogenic role in IBS, perhaps through their metabolic activity, this provides a target for new therapies and an intermediate phenotype by which to assess them.

## Introduction

IBS affects 10%–15% of the population worldwide.^1^ It impacts quality of life^2^ and incurs significant health economic cost.^3^ The pathophysiology of IBS includes changes in visceral nerve sensitivity,^4^ intestinal permeability^5^ and psychological factors.^6^ Several lines of evidence suggest the gut microbiome as a key aetiological factor in IBS. For example, there is a sixfold increased risk of developing IBS following an episode of infective gastroenteritis,^7^ probiotics and dietary intervention can reduce the symptoms^8 9^ and faecal transplantation has reported efficacy in treating IBS.^10^ Recent studies using 16S ribosomal RNA profiles have suggested an altered gut microbiota in IBS subjects compared with controls. Although the findings of earlier studies vary significantly, recent studies more consistently indicate a reduction in Bacteroidetes in IBS cases versus controls.^11–13^ However, the way in which the gut microbiota and IBS symptoms are linked mechanistically remains poorly understood.

IBS symptoms can be treated with low-fibre diets to reduce the colonic microbial fermentation that produces hydrogen and methane, leading to bloating.^9^ More recently, diets avoiding fermentable oligosaccharides, disaccharides, monosaccharides and polyols (FODMAPs) have demonstrated efficacy.^14–17^ The mechanisms are debated,^18^ but potentially involve modulation of microbiota composition and metabolite production.^19^ The low FODMAP diet is challenging for many patients to follow, often requiring increased time preparing meals, recipe adaptation and fewer options for convenience foods. Its long-term consequences on health are unknown. Thus, there is a recognised need to better understand how low FODMAP diets work,^20^ and ideally identify biomarkers that predict response.

In order to accurately link changes in gut microbiota structure with diet, including low FODMAP diets, detailed taxonomic profiling and quantification of microbial abundance is required. The gut microbiota of healthy adults is diverse, dominated by hundreds of bacterial species from the Bacteroidetes and Firmicutes phyla, with fewer species from Actinobacteria and Proteobacteria.^21^ It is shaped by diet and impacts immunity, metabolism and cognition.^22 23^ While 16S rRNA studies have provided valuable insights into the gut microbiota and IBS, they cannot achieve taxonomic resolution to species level. Techniques of microbial culture and metagenomic sequencing now enable detailed taxonomic and functional characterisation.^24^

The aim of the present study was to identify a biomarker of response to the low FODMAP diet and gain insights into microbial changes underlying treatment success using high-resolution metagenomic and functional analysis of subjects with IBS and household controls before and while on a low FODMAP diet.

## Materials and methods

### Subjects

A prospective single centre case–control study recruited participants from 2016 to 2019. We included adults (18–68 years of age) meeting the Rome IV criteria^25^ for diarrhoea-predominant or mixed type IBS (IBS-D and IBS-M, respectively) with respective household controls. Subjects were recruited from outpatient clinics at Cambridge University Hospital in the UK and via a social media campaign.

We excluded cases with other GI diseases, pregnancy, those already following a restrictive diet (including those already on a low intake of FODMAPs), and those taking probiotics or who had taken medications within 1 month that could potentially modify the gut microbiota such as antibiotics, proton pump inhibitors, colonoscopy bowel preparation or metformin.^26^

**Figure 1:**
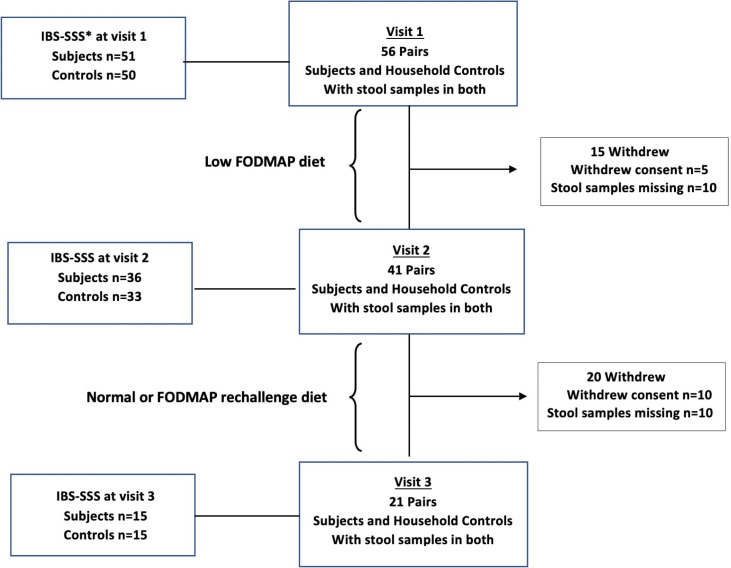
Flowchart for IBS microbiome study: number of pairs of IBS subjects and each of their household controls providing stool samples at visits 1–3. FODMAPs, fermentable oligosaccharides, disaccharides, monosaccharides and polyols; *IBS-SSS, IBS Severity Scoring System.

Study procedures are summarised in figure 1. Participants were assessed for suitability by a consultant and a dietitian. Three subsequent study visits were supervised by one of two senior gastroenterology dietitians trained and experienced in the administration of the low FODMAP diet. Seven-day food diaries (documenting the preceding week’s dietary intake) were collected from all participants and symptom severity scores captured using the IBS Severity Scoring System (IBS-SSS).^27^ Diet FODMAP scores were assessed using a previously published qualitative method^28^ as described in the supplemental materials (the FODMAP scores section).

### Stool samples

Participants and their household controls were asked to provide a stool sample at visit 1 while on their usual diet, after 4 weeks on a low FODMAP diet (at visit 2) and 12 weeks following FODMAP rechallenge in subjects with IBS improving on the diet (to identify individual FODMAP triggers), or a return to usual diet in subjects with IBS not improving with the diet and in all household controls (visit 3). Samples were sealed and immediately placed in the participant’s home freezer and then courier transferred on dry ice to the Wellcome Sanger Institute within 48 hours for storage at −80°C prior to processing. DNA was extracted using the MP Biomedicals FastDNA SPIN Kit for Soil.

### Metagenomic sequencing

To profile the taxonomic composition of the stool samples from cases and controls, we performed shotgun metagenomic sequencing using the Illumina Hi-Seq 4000 platform (read length 150 bp, 450 bp fragment size, average 12 million paired-end reads); total bacterial load was not assessed. Raw sequencing data were deposited under ENA Study Accession Number: ERP021923. Paired-end read files were classified using a Kraken2 bespoke database made of 2754 high-quality human GI genomes (online supplemental table 1) from 784 species associated with the human gut microbiome downloaded from Human Gastrointestinal Bacteria Culture Collection,^24^ Culturable Genome Reference^29^ and National Center for Biotechnology Information (NCBI). The quality of reference genomes was assessed using CheckM,^30^ and we kept assemblies with >90% completeness and <5% contamination. Bracken^31^ was applied to obtain refined species-level metagenomic profiles. An average of 10.2 million sequencing reads were classified at species rank by our platform, corresponding to a read classification rate of 86% (online supplemental figure 1). No difference in assigned read counts was observed between cases and controls (paired Wilcoxon p=0.7). Statistical analysis was performed using R language.^32^ Taxonomic profiles were normalised using centre log ratio (CLR) transform^33^ after estimating zero values using the *cmultRepl* function from *zCompositions* R package.^34^

10.1136/gutjnl-2021-325177.supp13 Supplementary data

10.1136/gutjnl-2021-325177.supp1 Supplementary data

For an unsupervised analysis to identify subpopulations of IBS cases, we applied a k-means clustering algorithm to CLR-transformed taxonomic profiles from baseline IBS case samples only. The optimal *k* value was obtained by maximising the average silhouette score using the *fviz_nbclust* function from the *factoextra* R package^35^ which evaluates the clustering quality for values between *k*=1–10. Additional clustering analyses were performed using only the household control samples and also with the combined samples for cases and controls.

A comparison of alpha diversity between groups was performed using a paired Wilcoxon signed-rank test. We used Aitchison distance^33^ which is the Euclidean distance of the CLR transformed profiles to estimate beta diversity between samples. The significance of beta diversity difference was estimated using Wilcoxon rank-sum test applied to pairwise Aitchison distances.

Associations between cluster assignment and clinical metadata were sought using Fisher’s exact test on the contingency table or Wilcoxon rank-sum test when appropriate (table 1). We tested for correlation between taxonomic/functional abundance and IBS cluster by applying generalised linear mixed models using MaAsLin2 software.^36^ To account for the non-independent samples, random effects were modelled by matching subject with IBS and household control, as well as longitudinal samples coming from the same individual. Raw data and source code for the analysis are available at http://github.com/kevinVervier/IBS.

**Table 1:** Baseline characteristics of the 56 subjects with IBS according to the cluster separation based on the differences in the microbiome

	Cluster 1 (n=28)	Cluster 2 (n=28)	P value
Female (%)	22 (79)	19 (68)	0.84
Age (mean±SD)	37.4±12.5	39.9±14.4	0.54
BMI (mean±SD)	29.4±7.7	26.5±5.4	0.08
IBSD (%)	12 (43)	11 (39)	1
IBSM (%)	16 (57)	17 (61)	1
Post-infectious IBS (%)	8 (28)	6 (21)	0.77
Median IBS-SSS	302 (n=24) (138–432)	249 (n=27) (79–439)	0.17
Median FODMAP score	8 (n=25) (5–12)	8 (n=27) (3–13)	0.58
Antidepressants (%)	6 (21)	3 (11)	0.48
PPIs/H2RAs (%)	1 (4)	0 (0)	1
Smokers (%)	4 (14)	1 (4)	0.36
Median alcohol intake (U/week)	3.5 (n=24) (0–28)	0.5 (n=26) (0–20)	0.13

Fisher’s test was applied on categorical variables (sex, IBSD, IBSM, post-infectious IBS, medications, smoking status), while Wilcoxon’s test was applied on continuous variables (age, BMI, IBS-SSS, FODMAP score, alcohol intake) to estimate statistical significance of the difference between groups.

BMI, body mass index; FODMAPs, fermentable oligosaccharides, disaccharides, monosaccharides and polyols; IBS-SSS, Irritable Bowel Syndrome Severity Scoring System.

Maximum-likelihood trees were generated using FastTree V.2.1.10^37^ with default parameters, and protein alignments were produced by GTDB-Tk V.1.3.0^38^ with the *classify_wf* function and default parameters. Trees were visualised and annotated with Interactive Tree Of Life (iTOL) V.5.^39^

### Functional metagenomic and genomic analysis

Functional profiling on each metagenome was conducted using HUMAnN3^40^ with default parameters to quantify MetaCyc pathways.^41^ Pathway enrichment was performed using MaAsLin2^36^ (threshold at q value <0.1). Enriched pathways were classified in broad categories using the MetaCyc database.

To identify the genes present in an enriched MetaCyc pathway in a reference genome, we first collected the protein sequence corresponding to each gene in each pathway from the Metacyc database and UniProt.^42^ BlastP^43^ was then performed for each of these protein sequences against a protein database based on 544 genomes (as a subset of the 2754 reference genomes) with a cut-off E value of 1e-10. This genome collection of 544 genomes includes 420 genomes (56 species) of IBS-associated bacteria representing cluster IBS^P^ and 124 genomes (34 species) of health-associated bacteria representing cluster IBS^H^ (see below for IBS^P^/IBS^H^ description). Gene enrichment was calculated using one-sided Fisher’s exact test with p value adjusted by Hochberg method.

## Results

### Cohort summary

The cohort is summarised in figure 1. Among cases, there was female predominance (73%) and IBS-M was the the most common subtype (59%). Fourteen cases (25%) reported symptom onset after an episode of gastroenteritis. The median IBS-SSS at baseline in the 56 cases was 272, with 45 cases (88.2%) scoring moderate (IBS-SSS >175 – n=25) or severe (IBS-SSS >300 – n=20), consistent with a typical population presenting to gastroenterology clinics.^28^ In controls, the median IBS-SSS score was 7.5 (range 0–196). Mean age of subjects was 38.7 (range 18–68) and controls 44.6 (range 18–74).

### Comparison of gut microbiota from IBS cases and household controls

Metagenomic sequencing was carried out on 234 stool samples followed by reference genome mapping of sequence reads.^24^ Our inclusion of household controls reduced confounding by environmental exposures (pets, prevailing diet, hygiene regime) and is important as gut microbes can frequently transmit between cohabiting humans.^44^ Indeed, we observed that samples coming from the same household had a more conserved microbiota composition compared with the overall variability between all cases and all controls (online supplemental figure 2C, Wilcoxon p=6.02E-05). To account for this potential confounder in subsequent analyses, we applied pairwise comparisons where possible.

10.1136/gutjnl-2021-325177.supp2 Supplementary data

We first focused on understanding the compositional variation in bacterial species to identify potential pathogenic imbalances in IBS case gut microbiomes. We compared baseline samples using the Chao1 index for the number of species (richness) and the Shannon index for the relative abundance of different species (alpha diversity). The richness was not lower in IBS cases (paired Wilcoxon p=0.12) (online supplemental figure 2A), but we did observe a lower alpha diversity in IBS cases compared with controls (paired Wilcoxon p=0.0092; online supplemental figure 2B). We also measured beta diversity between baseline microbiota samples using Aitchison distance and observed significantly more taxonomic variability within IBS cases compared with controls (online supplemental figure 2C, paired Wilcoxon p=1.3E-79).

### Stratification of IBS cases based on gut microbiota compositional subtypes

**Figure 2:**
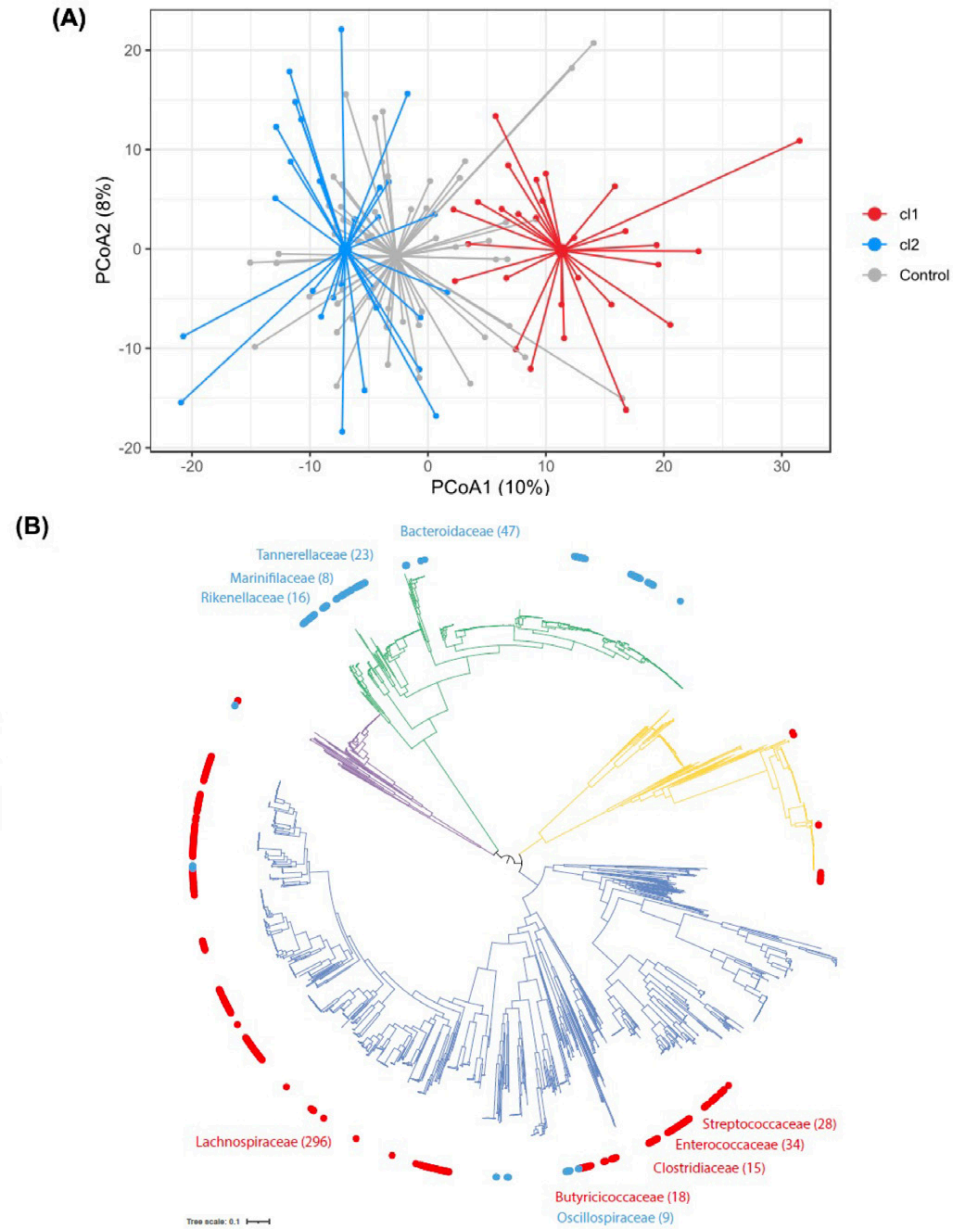
Analysis of diversity of microbiota profiles. (A) Beta diversity analysis of IBS cases and healthy controls: principal coordinate analysis for the two first components identifies two distinct clusters among cases, described as cluster 1 (cl1, red) and cluster 2 (cl2, blue). Overall dispersion of household controls is represented in grey. Variance explained by PC1: 10%, PC2: 8%. (B) Phylogenetic tree of 2754 human gut bacterial isolates generated using the 120 core genes. Outer circle distinguishes bacteria abundant in cl1 (red; n=420 genomes) and bacteria abundant in cl2 (blue; n=124 genomes). Top 5 prevalent families in each cluster are named. Branch colour distinguishes bacterial phyla belonging to Actinobacteria (yellow; n=363 genomes), Bacteroidetes (green; n=675 genomes), Firmicutes (dark blue; n=1562 genomes) and Proteobacteria (purple; n=154 genomes).

The high variability in diversity observed within baseline microbiomes from IBS cases warranted exploration of possible stratification by microbiome profile, to identify distinguishing signals that went undetected during our initial analysis. We therefore performed unsupervised data clustering—a hypothesis-free approach designed to identify microbiota subtypes—in baseline samples from the 56 IBS pairs. This analysis revealed optimal data separation being achieved on division into two distinct microbiota taxonomic clusters, with 28 cases assigned to each (figure 2A, online supplemental figure 3A, B).

10.1136/gutjnl-2021-325177.supp3 Supplementary data

This population stratification in two groups was replicated when considering cases paired with household controls (online supplemental figure 3C, D) but we could not observe a strong separation when clustering only the household controls (online supplemental figure 3E, F), suggesting that most of the signal captured in these clusters comes from variability in IBS cases. Principal coordinates analysis on the IBS cases alone captured a greater variability along the first two components (online supplemental figure 4, PC1: 14%, PC2: 9%) compared with cases plus controls. Microbiome composition in cluster 2 cases was significantly more similar to household controls compared with the cluster 1 cases using pairwise dissimilarity (Wilcoxon p=0.0037, online supplemental figure 5). Compared with the overall variability previously observed across all IBS cases, microbiota diversity within each cluster was more conserved (online supplemental figure 6, Wilcoxon p=2.2E-08). We found no significant difference in age, gender, body mass index (BMI), subtype of IBS, post-infectious IBS or concomitant medications between the two clusters (table 1). Baseline symptom severity scores appeared modestly higher in cluster 1 than cluster 2 (median IBS-SSS=302 vs 249), but this was not statistically significant (Wilcoxon p=0.17).

10.1136/gutjnl-2021-325177.supp4 Supplementary data

10.1136/gutjnl-2021-325177.supp5 Supplementary data

10.1136/gutjnl-2021-325177.supp6 Supplementary data

The number of bacterial species (richness) appeared modestly lower in cluster 1 cases compared with cluster 2 microbiomes (Wilcoxon p=0.033), but no such difference was observed between respective controls (Wilcoxon p=0.57) (online supplemental figure 7A). Cases and controls from the same cluster show comparable richness (paired Wilcoxon cluster 1 p=0.073, cluster 2 p=0.69). Shannon diversity (alpha diversity) was clearly lower in IBS cluster 1 compared with cluster 2 cases (p=0.0002), but this difference was not seen between respective controls (p=0.078) (online supplemental figure 7B). Cases from cluster 1 had a lower alpha diversity when compared with their household controls (paired Wilcoxon p=0.0029), while this was not observed for cluster 2 (paired Wilcoxon p=0.41). Overall, our findings suggest that fewer bacterial species are represented in cluster 1 case microbiomes and abundance profiles are skewed towards a smaller set of bacteria compared with cluster 2.

10.1136/gutjnl-2021-325177.supp7 Supplementary data

Read abundance analysis identified distinct differences between bacterial species in the two IBS subtypes at baseline (MaAsLin2 q value <0.1; online supplemental table 2). A total of 87 species were identified as significantly differentially abundant between the two IBS subtypes (56 up in cluster 1 and 31 up in cluster 2), but no such significant difference was observed between corresponding household controls. In IBS cluster 1, we observed a significant increase of bacteria from the Firmicutes phylum including known human pathogens (*Clostridium difficile*, *Paeniclostridium sordellii*, *Clostridium perfringens*, *Streptococcus anginosus*) (online supplemental figure 7C) and a significant depletion of multiple *Bacteroides* and *Parabacteroides* species (online supplemental figure 7D). Phylogenetic analysis showed a clear distinction between the dominant species from the Firmicutes phylum in cluster 1 and the dominant species from the Bacteriodetes phylum in cluster 2 (figure 2B). However, we did not observe a significant difference in abundance for these two phyla between groups (MaAsLin2 q-value: Firmicutes: 0.2, Bacteroidetes: 0.78), suggesting differences in a subset of species rather than an overall Firmicutes/Bacteroidetes imbalance.

10.1136/gutjnl-2021-325177.supp14 Supplementary data

Thus, we identified IBS subtypes with distinct microbiota signatures at baseline: cluster 1 contained lower bacterial diversity, was depleted in commensal species from the Bacteroidetes phylum and enriched in species from the Firmicutes phylum, including human pathogens; and cluster 2 was indistinguishable from healthy household controls. We refer to cluster 1 as IBS^P^ microbiome type for its pathogenic properties and cluster 2 as IBS^H^ microbiome type due to its similarity to healthy household controls.

### Enrichment of primary metabolism genes in gut microbiomes of IBS^P^ patients

**Figure 3:**
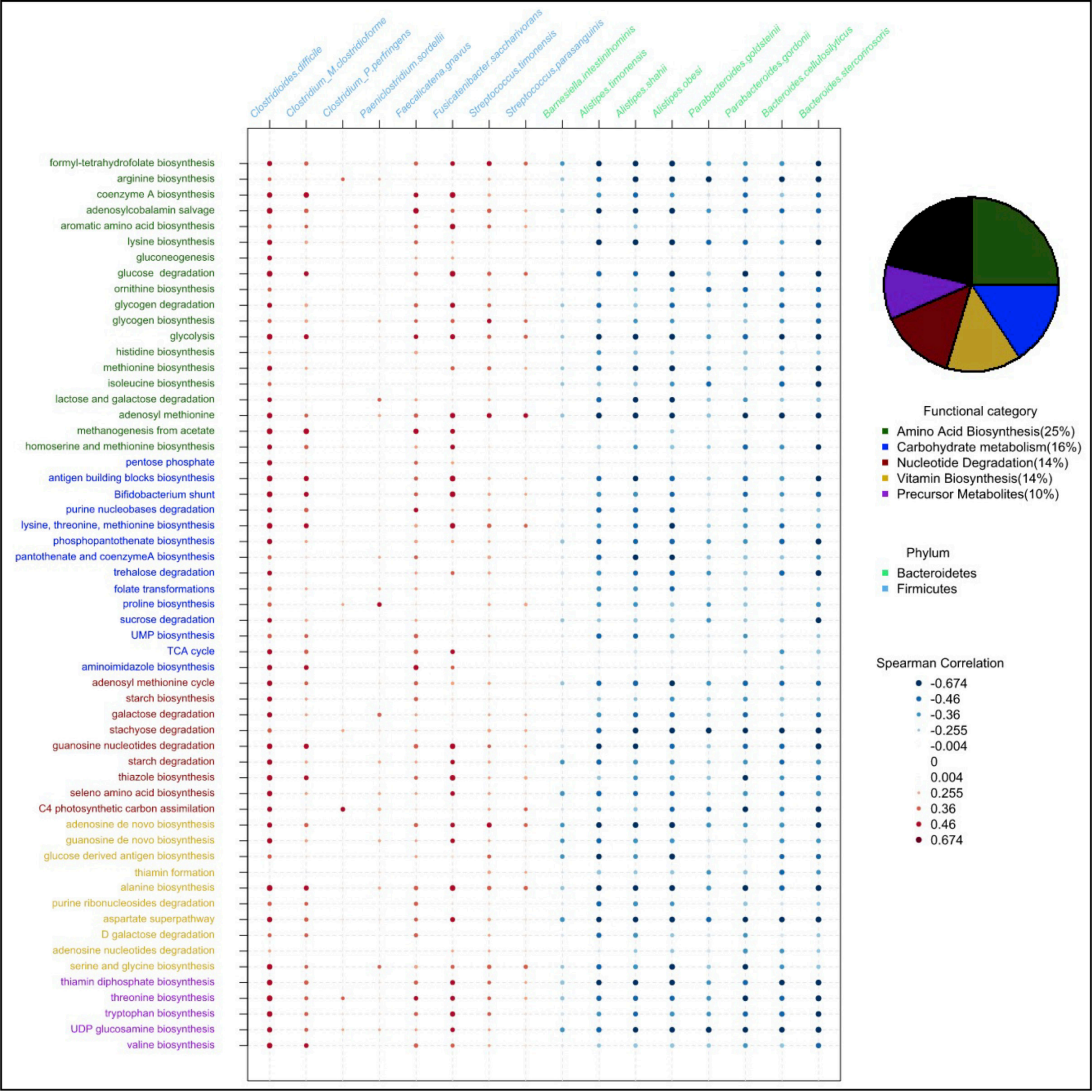
Functional and taxonomic characterisation of IBS^P^ subjects baseline microbiomes. Pie chart indicates the distribution of pathways identified as significantly enriched in IBS^P^ subjects at baseline and coloured according to their MetaCyc functional category. A selection of candidate pathways are represented in rows (coloured as in the pie chart). Species significantly different in abundance between IBS^P^ and IBS^H^ subjects are represented in columns and coloured by phylum (Bacteroidetes in green and Firmicutes in blue). For each combination of pathway and species, Spearman correlation on their respective abundance is reported (from strongly positive in red to strongly negative in blue).

Bacterial species from the Bacteroidetes and Firmicutes phyla are evolutionarily and physiologically distinct, and contribute different core functions to the gut microbiome. Therefore, we reasoned that the functional capacity of IBS^P^ microbiomes may contribute to IBS symptoms. To identify functional differences between the microbiomes of the two IBS subtypes, we performed an analysis of the functional capacity encoded in the metagenomes of baseline samples of IBS^P^ and IBS^H^ patients. This analysis was independent of the previous taxonomic analysis. We found a significant enrichment of 109 functional pathways and significant depletion of 13 functional pathways in IBS^P^ microbiomes compared with IBS^H^ microbiomes (online supplemental table 3). Further functional classification indicated that the majority of enriched pathways in IBS^P^ microbiomes (78.7%) could be classified to five major functional categories related to primary metabolism (figure 3). This signal was replicated in an analysis which included control samples, where 51 out of the 53 significant findings were already reported in the 112 pathways. It suggests that functional differences between IBS^P^ and IBS^H^ patients are not attributable to environmental/lifestyle factors.

10.1136/gutjnl-2021-325177.supp15 Supplementary data

Since amino acid biosynthesis (25%) and carbohydrate metabolism (15.7%) were the two major functional categories that separate IBS^P^ and IBS^H^ cases, we next performed a targeted functional enrichment analysis in IBS^P^ microbiomes at the species level. For amino acid biosynthesis, this identified significant enrichment of genes involved in biosynthesis of tryptophan, threonine and histidine (online supplemental figure 8). Equivalent analysis of carbohydrate metabolism identified significant enrichment of genes involved in lactose metabolism, fructose metabolism and trehalose metabolism, and biosynthesis of two short chain fatty acids (SCFA): butyrate and propionate (online supplemental figure 9).

10.1136/gutjnl-2021-325177.supp8 Supplementary data

10.1136/gutjnl-2021-325177.supp9 Supplementary data

Our results suggest specific functions involved in amino acid biosynthesis and metabolism of simple dietary sugars are distinct features in bacteria of the IBS^P^ cluster at baseline, which are under-represented in bacteria of the IBS^H^ cluster. Correlating the compositional (figure 2B) and functional (figure 3) features identified a subset of candidate species associated with the IBS^P^ cluster (figure 3) and enriched in significant pathways. A strong positive correlation was observed between the abundance of these pathways and abundance of the bacterial species with known pathogenic capabilities (*C. difficile*, *P. sordellii*, *C. perfringens*) and a pathobiont associated with UC (*Faecalicatena gnavus,* previously named *Ruminococcus gnavus* 45). Commensal species depleted in IBS^P^ patients did not encode these pathways.

### Low FODMAP dietary intervention corrects IBS^P^ microbiomes

**Figure 4:**
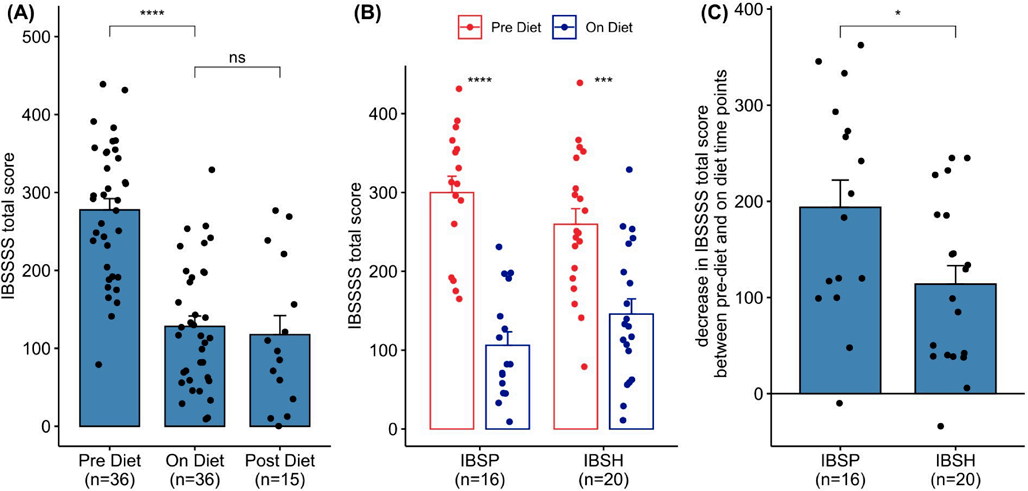
Clinical response in 36 subjects undergoing dietary intervention and providing IBS-SSS. (A) Response for combined IBS^P^ and IBS^H^ subjects pre-diet and on-diet also includes IBS-SSS in 15 subjects at visit 3. (B) Response pre-diet and on diet according to the microbiota cluster pre-diet. (C) Change in IBS-SSS from pre-diet value to on diet value for patients in each cluster. Paired Wilcoxon’s test was used to estimate statistical significance of the difference between groups (****p<0.0001, ***p<0.001, *p<0.05, ns: p>0.05). Bar height shows mean value, error bars show SE. IBS-SSS, Irritable Bowel Syndrome Severity Scoring System.

A total of 41 IBS cases and their household controls followed a low FODMAP diet for 4 weeks and provided a stool sample while on the diet. There was no significant difference in FODMAP scores at baseline or on the diet between IBS^P^ and IBS^H^ clusters, and as expected, the scores fell significantly for each cluster on diet (online supplemental material 1—FODMAP scores, Wilcoxon p=<0.00001). There was a significant reduction in the IBS-SSS on the low FODMAP diet (mean IBS-SSS pre-diet=278, on diet=128) (figure 4A). This was observed both in patients harbouring IBS^P^ and IBS^H^-type microbiomes (figure 4B) but the difference in degree of response was more pronounced in IBS^P^ patients (ΔIBS-SSS in IBS^P^=194 vs IBS^H^=114; Wilcoxon p=0.02) (figure 4C). Response rates defined by a fall in IBS-SSS >50 points^27^ between visits 1 and 2 followed the same trend but did not reach statistical significance (14/16 (87.5%) of IBS^P^ vs 13/20 (65%) of IBS^H^; χ^2^ test p=0.12).

10.1136/gutjnl-2021-325177.supp16 Supplementary data

IBS-SSS remained lower than at visit 1, 3 months after the completion of the low FODMAP diet (mean IBS-SSS post-diet=117), but the amount of FODMAP data obtained at this time point did not allow analysis with adequate power.

**Figure 5:**
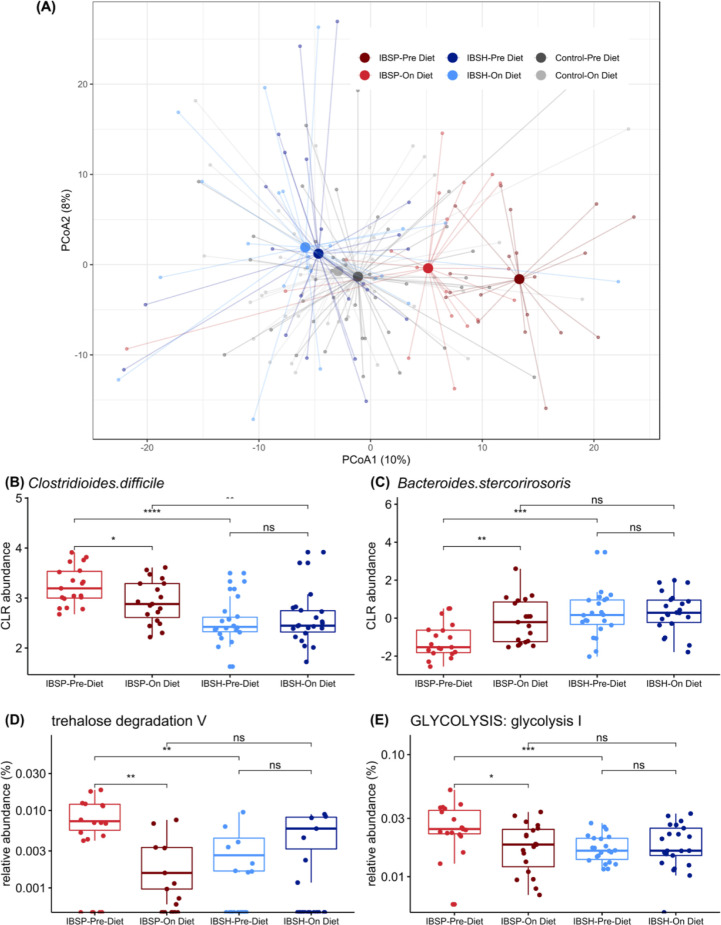
Microbiome beta diversity before and during diet intervention. (A) Principal coordinate analysis of IBS cases separated into two clusters showed a diet-triggered shift in IBS^P^ (red) only—not seen in IBS^H^ subjects (blue) or healthy controls (grey). (B, C) Impact of diet intervention on taxonomic abundance. Linear mixed models identified differentially abundant species between IBS^P^ and IBS^H^ cases pre-diet and on diet. Centre log ratio (CLR) transformed abundances for representative species are shown. (B) Pathobiont species, such as *Clostridium difficile*, become less abundant in IBS^P^ during diet intervention. (C) Members of *Bacteroides* genus become more abundant in IBS^P^ during diet intervention. (D, E) Impact of diet intervention on pathway abundance. Relative abundances for representative pathways are shown. (D) Degradation of the fermentable disaccharide trehalose became less abundant in IBS^P^ during diet intervention. (E) Glycolysis became less abundant in IBS^P^ during diet intervention. Wilcoxon’s test was used to estimate statistical significance of the difference between groups (****p<0.0001, ***p<0.001, **p<0.01, *p<0.05, ns: p>0.05). Box and whiskers show median and IQR.

Comparison of taxonomic profiles between baseline (pre-diet) stool samples and those obtained while on the low FODMAP diet for 4 weeks revealed a significant shift in the microbiota composition of IBS^P^ cases but not IBS^H^ cases nor healthy controls (figure 5A). Compared with the differences seen between IBS^P^ and IBS^H^ at baseline, beta diversity analysis showed the microbiome profiles from IBS^P^ cases became more similar to those seen in IBS^H^ cases and healthy controls while on the low FODMAP diet. This was apparent as a decreased variability in microbiome composition within all IBS cases (IBS^P^+IBS^H^ combined) on diet compared with pre-diet (online supplemental figure 10, paired Wilcoxon test p=1E-19). It was also evident that the diet produced a greater shift in microbiota composition in IBS^P^ compared with IBS^H^, with a bigger distance between sample profiles from the same case at the two timepoints (baseline and on-diet) (online supplemental figure 10, paired Wilcoxon test p=0.03).

10.1136/gutjnl-2021-325177.supp10 Supplementary data

Diet intervention shifted the taxonomic composition of IBS^P^ cases by increasing *Bacteroides* levels (*B. cutis, B. stercorirosoris*), and decreasing pathobiont levels (including *C. difficile*, *Streptococcus parasanguinis*, *Paeniclostridium sordellii*) towards those seen in IBS^H^ (figure 5B, C) and household controls (online supplemental figure 11). The functional profile of the IBS^P^ microbiome was also impacted by the diet intervention, for example, producing a decrease in degradation of the disaccharide trehalose (figure 5D) and a decrease in glycolysis to levels comparable to those in IBS^H^ patients and healthy controls (figure 5E).

10.1136/gutjnl-2021-325177.supp11 Supplementary data

After the low FODMAP diet ended, participants returned to a normal diet, although with cases limiting foods identified as triggering their symptoms (online supplemental material 1, the Dietary intervention section). Although the numbers available for 3-month follow-up limit the strength of the conclusions at this timepoint, there appeared to be no significant shift in the microbiota diversity of the cases in the two clusters compared with while on full dietary restriction (Wilcoxon p=0.12, online supplemental figure 12) and no significant change in the abundance of any bacterial taxa between these timepoints. Thus, the shift in the IBS^P^ microbiota to a heathy profile appeared stable for at least 3 months and correlated with continuing symptomatic well-being (figure 4A).

10.1136/gutjnl-2021-325177.supp12 Supplementary data

## Discussion

We defined two gut microbiome subtypes in IBS cases with distinct signatures based on species and encoded microbial functions, and differential clinical responses to a low FODMAP diet. Although the early microbiome literature is rather inconsistent regarding taxa implicated in IBS and the presence of subtypes,^11^ possibly reflecting clinical heterogeneity, choice of controls and methodology among other factors, our work is congruent with the observations of more recent studies^12 13^: Jeffery *et al* used shotgun 16S rRNA gene microbiome profiling and metabolomics to provide evidence of IBS microbiome subtypes, identifying Lachnospiraceae species and enrichment in amino acid biosynthesis. Not only do our results replicate this stratification within a larger IBS cohort, but being based on shotgun metagenomics data they benefit from both greater taxonomic resolution— identifying an increase in selected Firmicutes species and depletion of Bacteroidetes species in one subgroup—and the ability to analyse the functions encoded in the microbiome. Furthermore, the dietary intervention allowed us to characterise the clinical responses of each patient subtype; and inclusion of household controls, following the same dietary intervention, was a unique feature of our study designed to correct known confounding environmental effects.^46^

We refer to the IBS microbiome subtypes as IBS^p^ (pathogenic) and IBS^H^ (healthy). Overall, while recognising the likely contribution of a placebo effect, 75% of IBS cases in our study improved on a low FODMAP diet as measured by a decrease in IBS-SSS of more than 50 points; but higher degrees of symptom response were seen in cases with IBS^P^ compared with IBS^H^ microbiomes. IBS^P^ microbiomes were notably different from those of IBS^H^ cases and healthy household controls, with an enrichment of distinct bacterial species and gene families seen in IBS^P^ that allows us to propose potential pathogenic mechanisms.

Within the dysbiotic IBS^P^ microbiomes, we saw a significant enrichment of a broad range of evolutionarily distinct Firmicutes species, including known human pathogens (*C. difficile*, *C. sordellii* and *C. perfringens*), a pathobiont associated with UC (*Faecalicatena gnavus,* previously named *R. gnavus* 45) and known gut species not previously identified as human pathobionts (*C. clostridioforme* and *Fusicatenibacter saccharivorans*). Interestingly, we also saw an enrichment in IBS^P^ microbiomes of the lactic acid bacteria *Streptococcus parasanguinis* and *Streptococcus timonensis* that are usually found in the oral cavity.

At a functional level, IBS^P^ microbiomes were enriched in genes and pathways involved in metabolising carbohydrates. This could lead to increased anaerobic glycolysis and associated carbon dioxide, hydrogen and methane production in individuals with these microbiomes, with consequent increased gut distension contributing to increased symptoms. Among simple sugars recognised as FODMAPs are lactose and fructose so our functional microbial analysis provides a list of candidate bacteria for further analysis (online supplemental figure 9). The disaccharide trehalose is not a FODMAP, but if inaccessible to the brush border enzyme trehalase, for example, due to food-residue consistency, it may enter the colon to exert FODMAP-like properties through fermentation. Although not specifically prohibited, there is crossover between foods excluded on the low FODMAP diet and foods high in this disaccharide such as mushrooms. Of note, specific lineages of *C. difficile* have evolved to avidly metabolise trehalose and in so doing increase their abundance^47^—one route by which specific ‘pathogenic’ bacterial species could trigger IBS symptoms. Trehalose could trigger IBS symptoms by fuelling the growth of specific ‘pathogenic’ bacterial species.

Microbial metabolism of hexoses derived from FODMAP carbohydrates produces pyruvate by anaerobic glycolysis in the gut. Pyruvate is a key metabolite that feeds into SCFA production.^48^ Our pathway analysis (online supplemental figure 9) predicts that several bacterial species enriched in IBS^P^ microbiomes contain genes for converting pyruvate to butyrate (classical pathway) and/or propionate (acrylate pathway).^49^ Butyrate and propionate are major metabolites in the colon that bind to GPR receptors 41, 43 (propionate) and 109A (butyrate): these SCFAs regulate tryptophan hydrolase gene transcription in enterochromaffin cells facilitating the production of 5-hydroxytryptamine (5HT) from tryptophan; 5HT is postulated as a key agent in the production of IBS symptoms.^50 51^ Moreover, in IBS^P^ microbiomes, we observed an enrichment of genes for tryptophan biosynthesis which would facilitate this mechanism.

We also found enrichment in IBS^P^ microbiomes for the genes coding for the biosynthesis of amino acids including histidine, arginine, ornithine, tryptophan, alanine and threonine (online supplemental figure 8). Interestingly, Lee *et al* 52 found elevated levels of threonine, tryptophan and phenylalanine, as well as amino acid metabolites cadaverine and putrescine, in stool samples of patients with IBS, providing direct evidence of altered amino acid metabolism. Histidine is a precursor to histamine, implicated in the generation of IBS symptoms following its release from mast cells; histamine can itself also activate these cells.^53^

Although we detected higher levels of specific pathogens in IBS^P^ microbiomes, we have no evidence to suggest they are causing IBS symptoms through known toxin virulence factors. Instead, the data suggest an enrichment of primary metabolic pathways in diverse Firmicutes species. Our analysis indicates a potential for increased production of amino acids; and SCFA through metabolising FODMAP carbohydrates. It is possible that such metabolites and their derivatives could be noxious at high levels within the colon, or be pathological if produced within the wrong intestinal niche, a type of metabolic virulence, leading to IBS symptoms. One key finding from our work is that IBS^P^ and IBS^H^ microbiomes have distinct bacterial community responses to low FODMAP dietary intervention, providing a basis to define a mode of action. Thus, it is possible that removal of the eliciting dietary component starves the pathobionts leading to reduction in their growth and metabolism and a consequent decrease in symptoms, accompanied by an expansion of commensal or symbiotic species leading to a health-associated microbiome. The evidence associating diet, the microbiome and symptoms in IBS^P^ is compelling but studies following the introduction of candidate organisms into an animal model are needed to prove the relationship is causal.

Although the number of case/control pairs (n=21) who provided follow-up samples at 12 weeks after rechallenge with FODMAPs was relatively modest, and some continued to exclude specific FODMAP-containing foods, it was interesting to note that both their symptoms (figure 4A) and microbiomes (online supplemental figure 12) remained notably stable. This corroborates and perhaps helps to explain the durable benefit that can be seen from a low FODMAP diet.

We observed a differential response of IBS^P^ and IBS^H^ microbiome subtypes to the low FODMAP diet, suggesting that some gut microbiomes are more influenced by dietary interventions. Based on our analysis, it is not obvious how or whether IBS^H^ microbiomes contribute to IBS symptoms since they are indistinguishable from household control microbiomes and did not significantly alter in response to the low FODMAP diet. That symptoms in IBS^H^ cases still improved somewhat on FODMAP reduction suggests either that the response is linked to a non-bacterial component of the microbiome, such as viruses, or is unconnected mechanistically to the microbiota, perhaps instead reflecting a direct effect of dietary constituents and their metabolites on gut neuronal function or osmotic load or indeed simply a placebo effect in this group.

The presence of microbially defined IBS subtypes with differing responses to intervention has been suggested by some previous studies. In a recent faecal microbiota transplantation study, patients with IBS responding to the treatment showed enrichment in taxa such as *Bacteroides*, positively correlated with IBS-SSS decrease, as well as a drop in pathobionts such as Streptococci.^54^ In other studies, stool microbial profiles assessed by a commercial kit correlated with differing responses to a low FODMAP diet^55^; and the profile of faecal volatile organic compounds, postulated as reflecting microbiome differences, predicted response to a low FODMAP diet or probiotics.^56^

Our study does have limitations. The sample size was relatively modest: the strict inclusion criteria, the restriction of concomitant medications and the required participation of household controls needing to follow the low FODMAP diet hindered recruitment. Dietary information was limited to the last week of the interventional phase of the low FODMAP diet: participants could have been tempted to follow a more rigorous diet on the week they had to report their dietary intake. With the design of the study, it was impossible to exclude other factors, apart from diet, that could have impacted the benefit observed, including the psychological impact of being assessed within a research study, the placebo effect that has been described in other studies, and referral bias. Our findings of distinct IBS clusters based on microbiome profiles, the shift on the low FODMAP diet and the clinical responses, should be validated in other populations from different geographical distributions and exposed to different dietary habits.

The identification of a microbial signature ‘biomarker’ that correlates with improved response to a low FODMAP diet may, if validated, allow better stratification and selection of patients likely to benefit from the diet. In IBS^P^ subjects, there is the prospect to consider therapeutic strategies that manipulate the microbiota in the same direction and achieve the same symptomatic improvement but without the need to undergo the same stringent dietary restrictions. Further, closer study of the implicated microbes may give the opportunity to better understand the interaction between diet, microbiota, metabolites and the human gut-brain axis that leads to the development of IBS symptoms in more than 10% of the world’s population.

## Data Availability

Data are available in a public, open access repository. Raw sequencing data are accessible under ENA Study Accession Number: ERP021923Processed data are submitted as supplementary materials.Source code and scripts necessary to replicate the analysis are submitted at http://github.com/kevinVervier/IBS.
