# Supplementary figures and images for: Two microbiota subtypes identified in irritable bowel syndrome with distinct responses to the low FODMAP diet
Source: Gut. 2021 Nov 22;71(9):1821–30. doi: 10.1136/gutjnl-2021-325177 (PMC9380505; doi:10.1136/gutjnl-2021-325177)

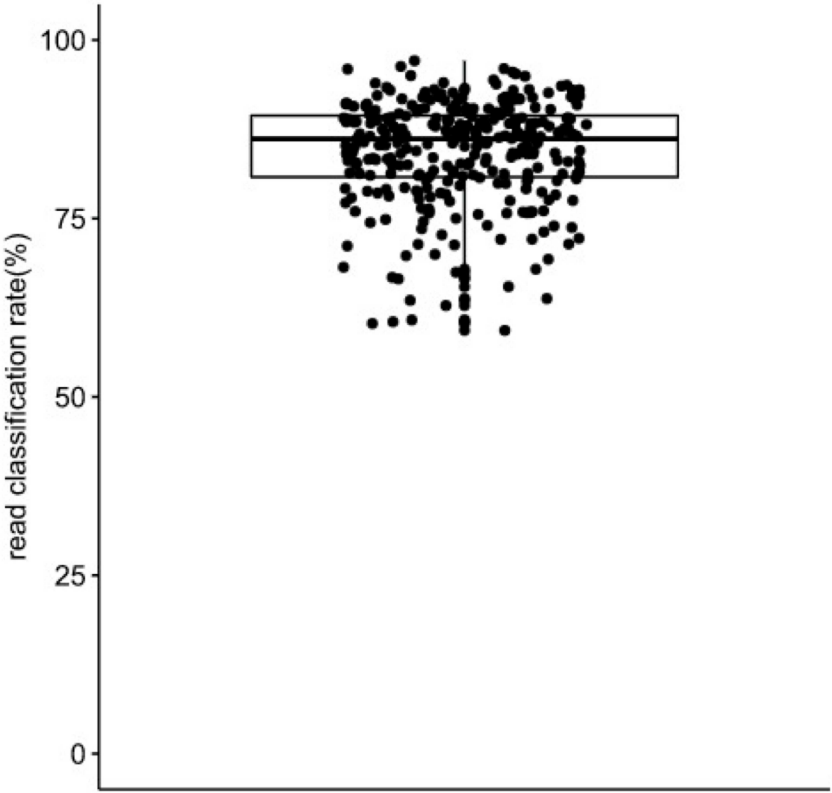

Supplement: Supplementary data [file gutjnl-2021-325177supp001.pdf]

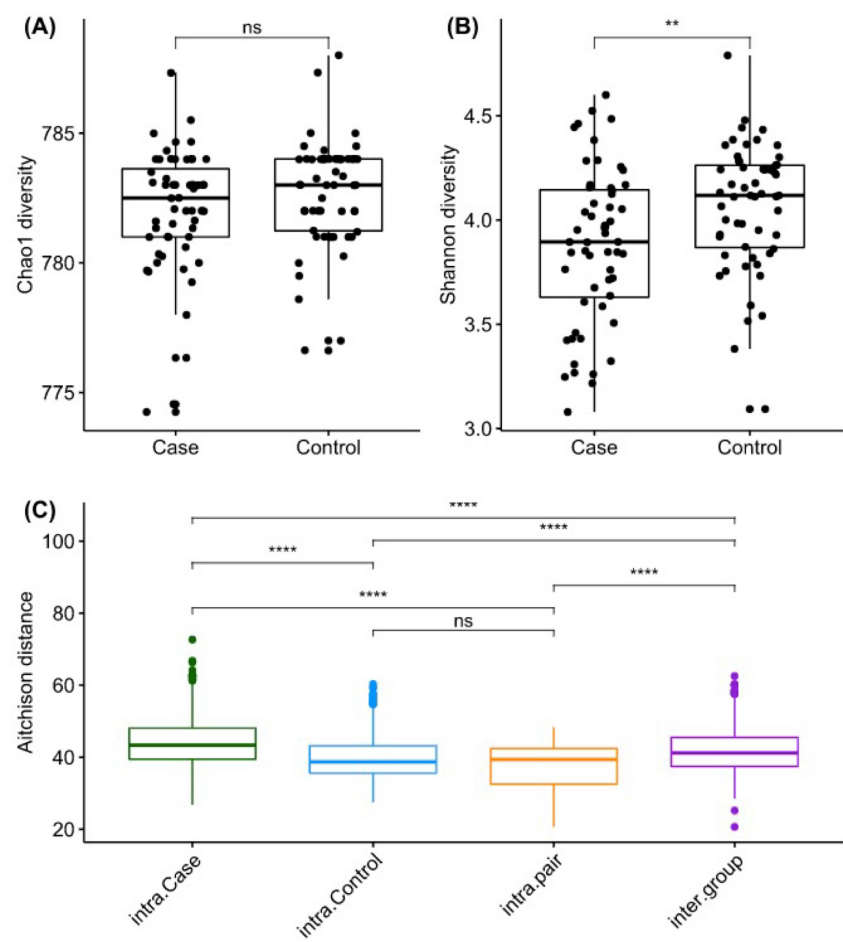

Supplement: Supplementary data [file gutjnl-2021-325177supp002.pdf]

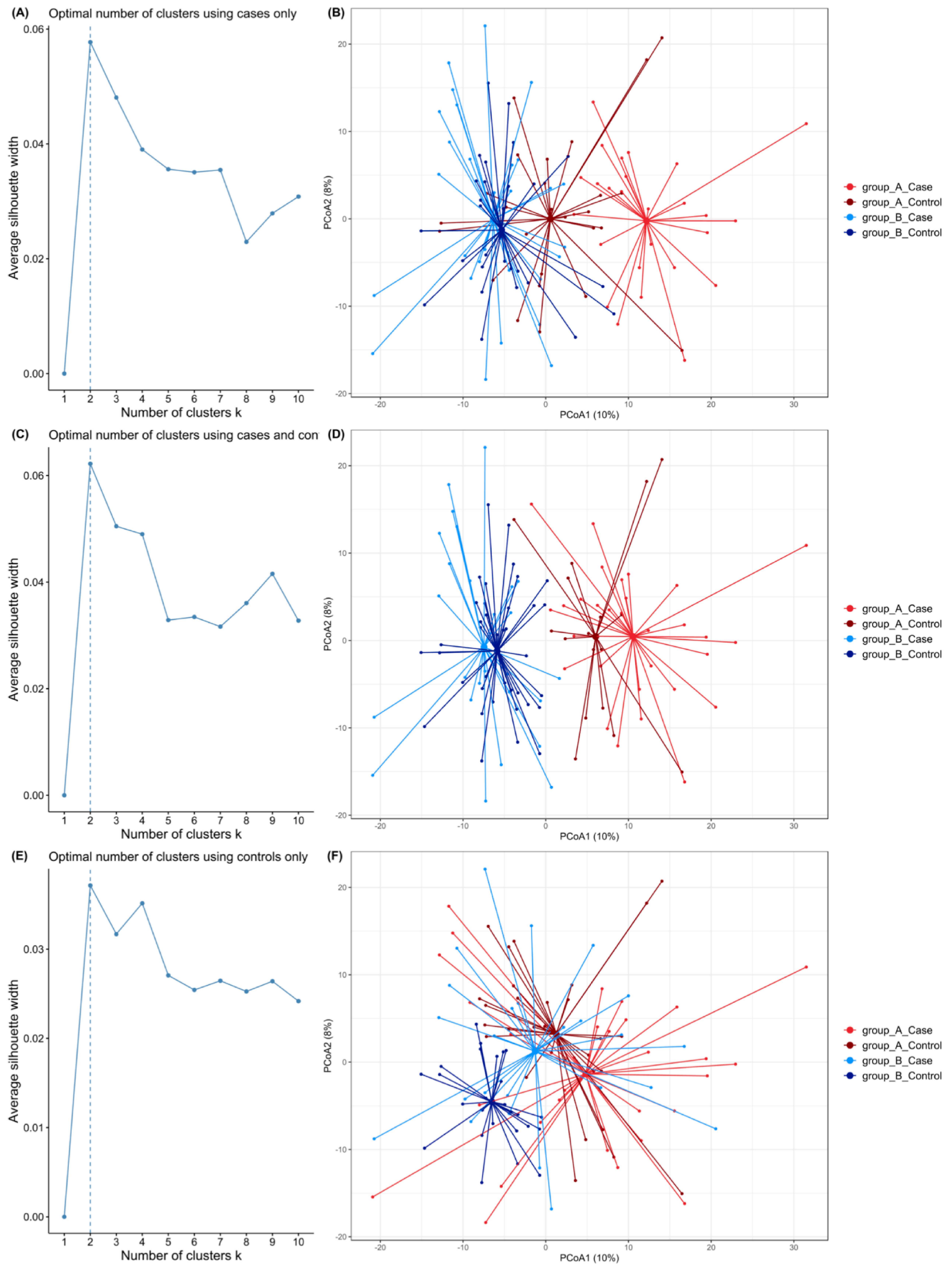

Supplement: Supplementary data [file gutjnl-2021-325177supp003.pdf]

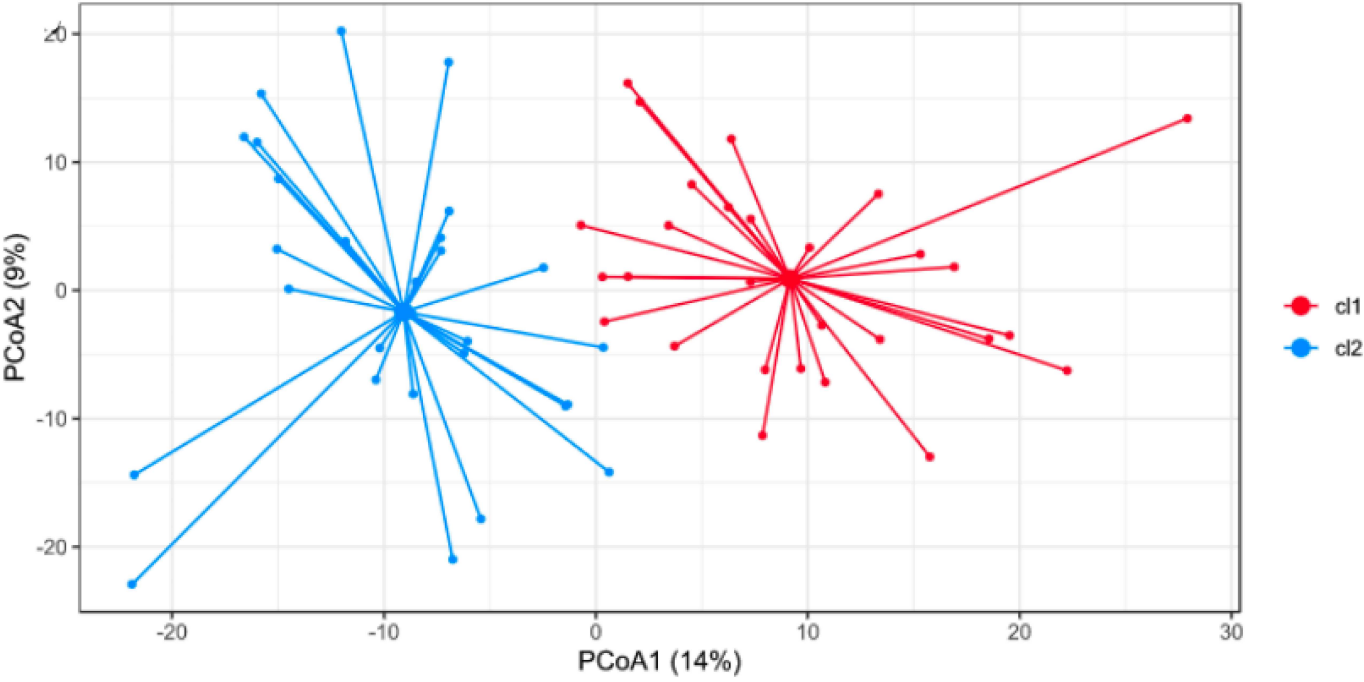

Supplement: Supplementary data [file gutjnl-2021-325177supp004.pdf]

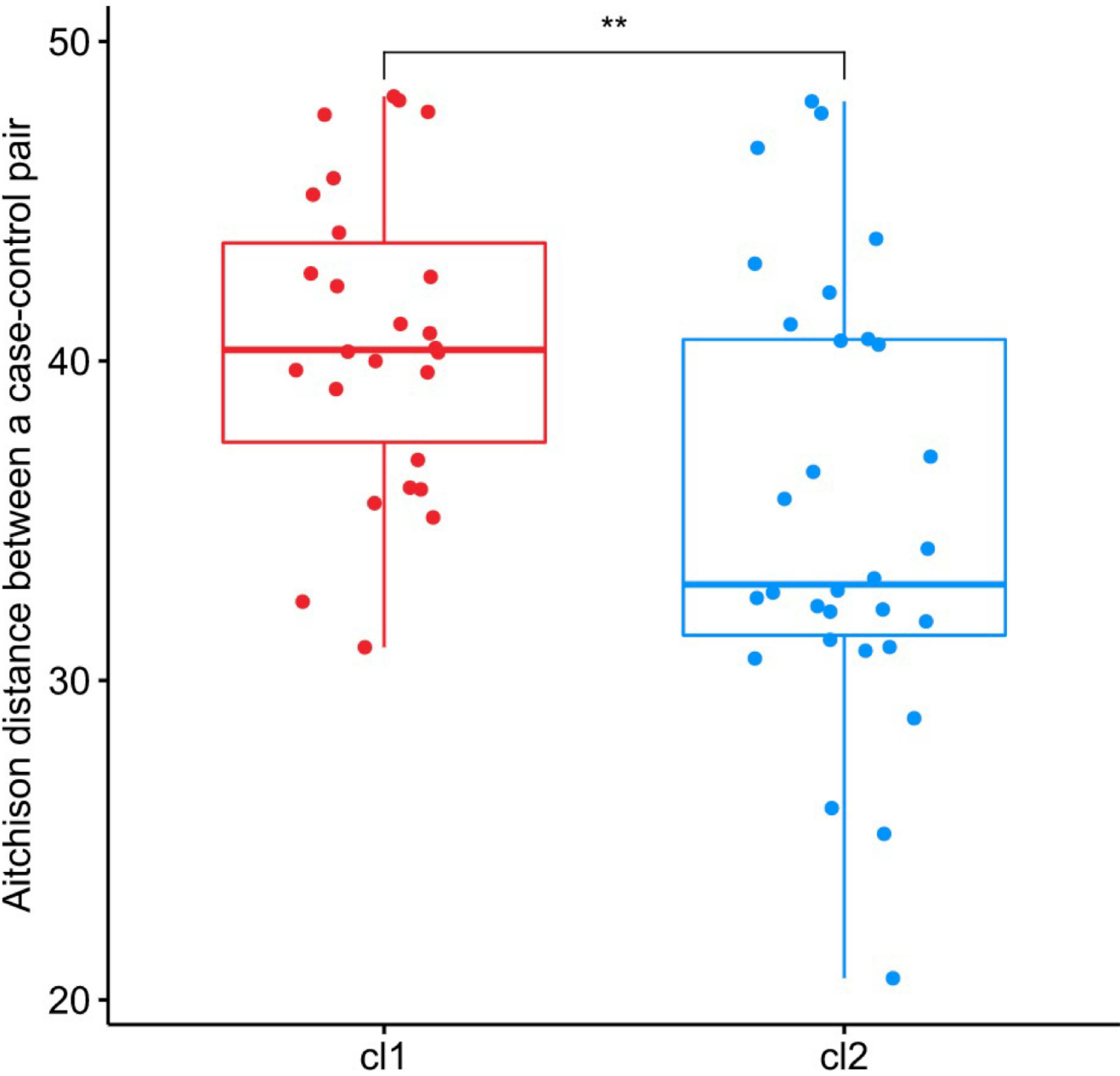

Supplement: Supplementary data [file gutjnl-2021-325177supp005.pdf]

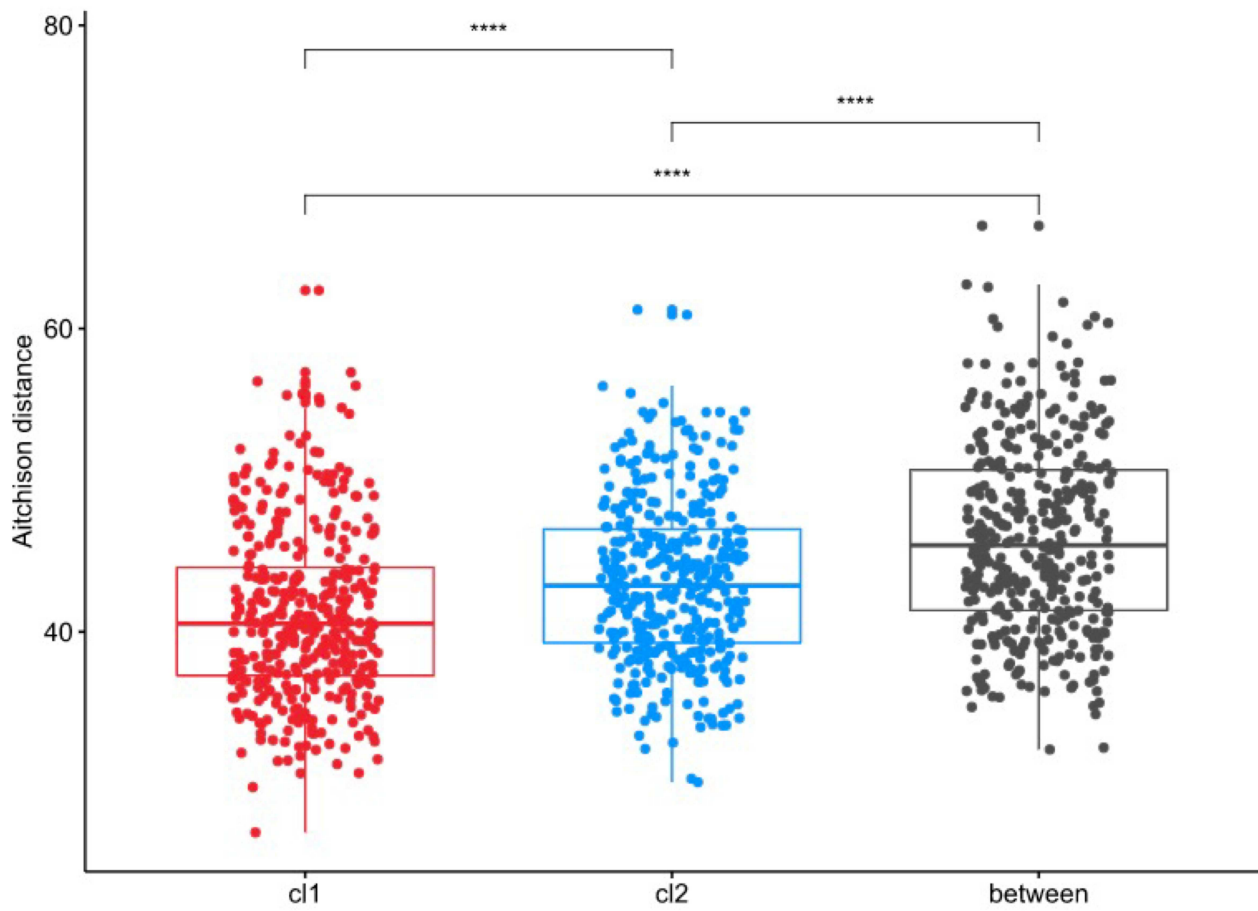

Supplement: Supplementary data [file gutjnl-2021-325177supp006.pdf]

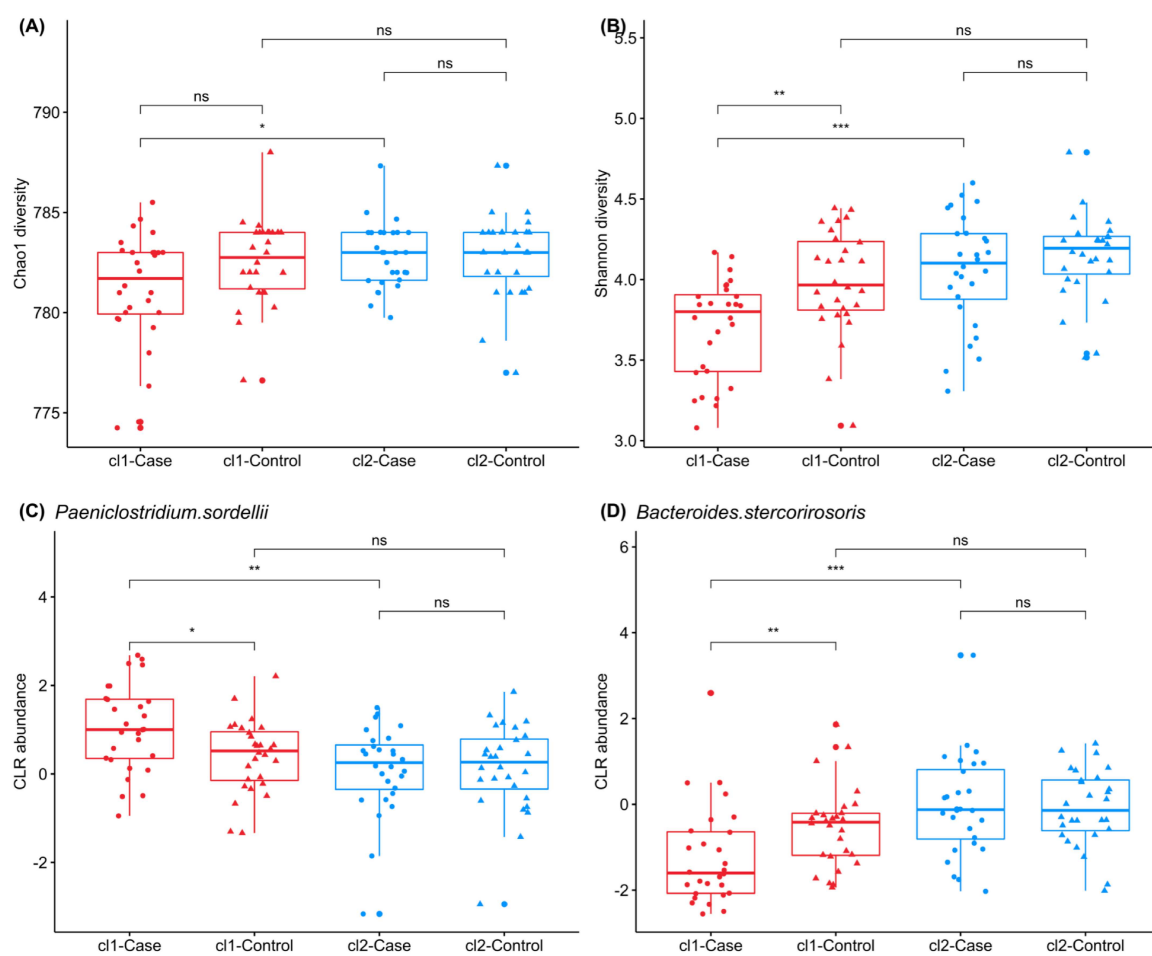

Supplement: Supplementary data [file gutjnl-2021-325177supp007.pdf]

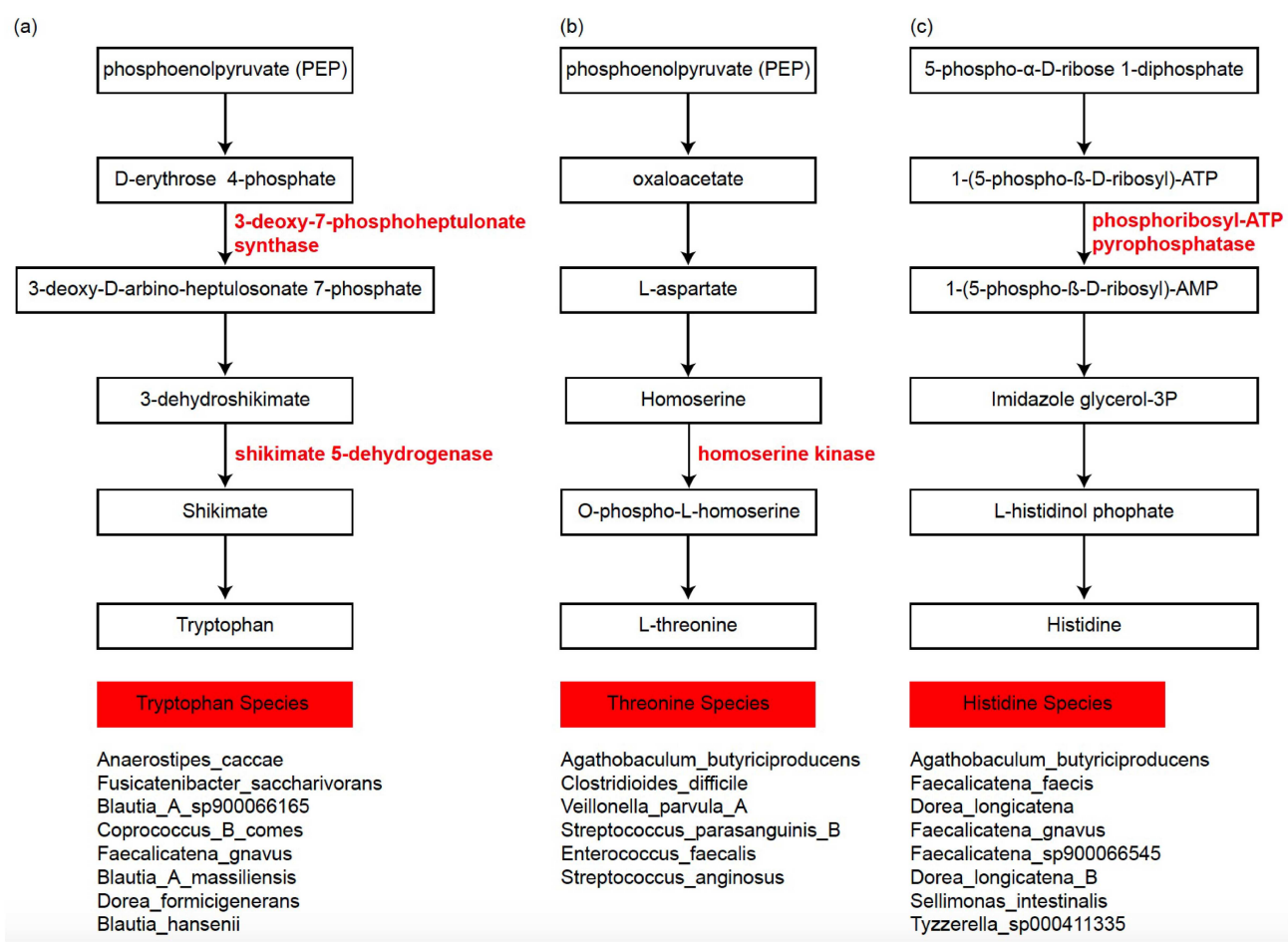

Supplement: Supplementary data [file gutjnl-2021-325177supp008.pdf]

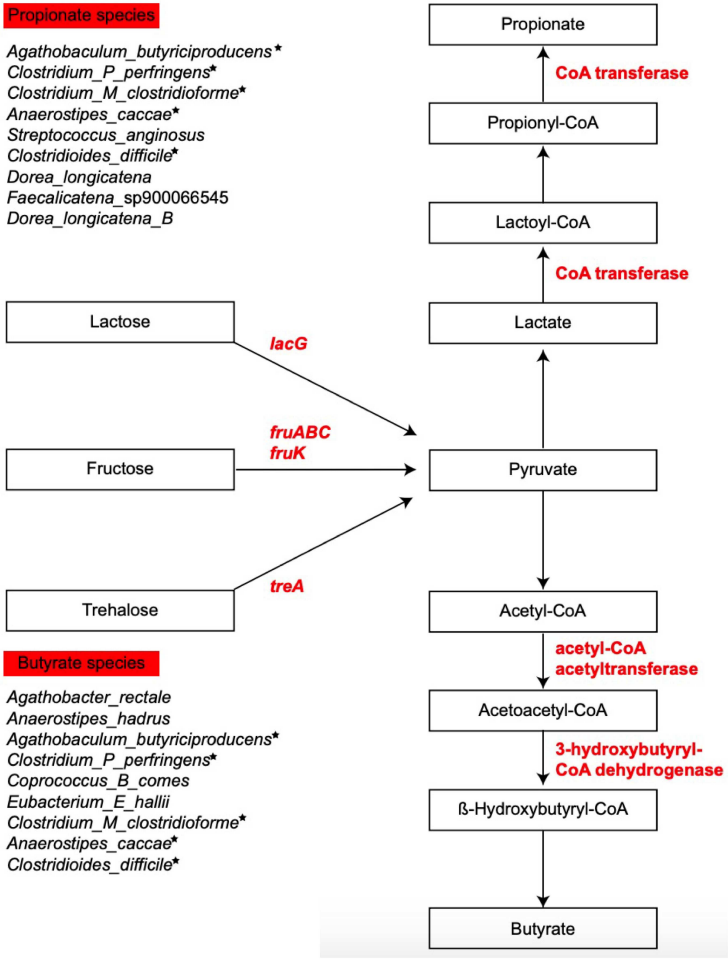

Supplement: Supplementary data [file gutjnl-2021-325177supp009.pdf]

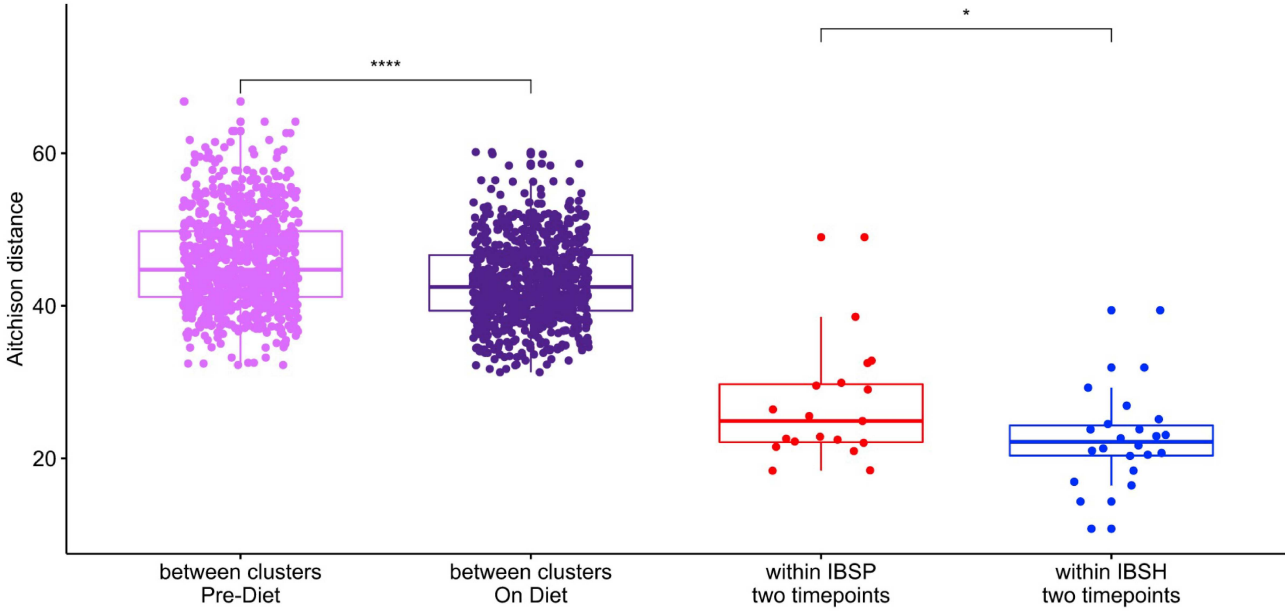

Supplement: Supplementary data [file gutjnl-2021-325177supp010.pdf]

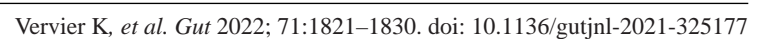

Supplement: Supplementary data [file gutjnl-2021-325177supp011.pdf]

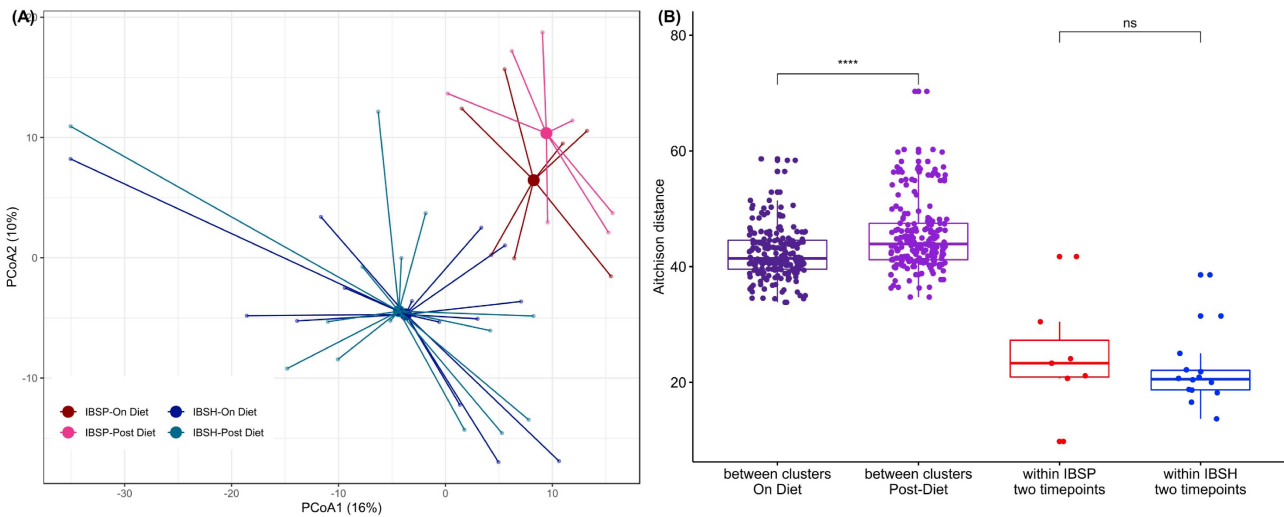

Supplement: Supplementary data [file gutjnl-2021-325177supp012.pdf]
