# Supplementary material for: Two microbiota subtypes identified in irritable bowel syndrome with distinct responses to the low FODMAP diet
Source: Gut. 2021 Nov 22;71(9):1821–30. doi: 10.1136/gutjnl-2021-325177 (PMC9380505; doi:10.1136/gutjnl-2021-325177)
Supplement: Supplementary data [file gutjnl-2021-325177supp016.pdf]

Supplementary Materials

Dietary intervention

At the first visit, baseline IBS symptom questionnaires and 7-day food diaries (documenting habitual intake) were collected from all participants and assessed by experienced GI dietitians. Cases who on initial assessment were already consuming a diet low in FODMAPs were not included in the study. Education was provided on how to follow the low FODMAP diet by a specialist dietitian with supporting written literature.

At visit 2, after 4 weeks on the low FODMAP diet, further IBS symptom questionnaires and 7-day food diaries were collected. Household controls, and cases who had not responded to the diet, were advised to return to their normal diet after 4 weeks on the low FODMAP diet. If IBS subjects had responded to the diet education was provided on the FODMAP re-introduction phase with supporting literature. Each subgroup of fermentable carbohydrate: GOS, lactose, fructose, sorbitol, mannitol plus high fructan containing foods: wheat, onion, garlic and leeks, was individually challenged in increasing amounts during a period of up to three days. If no symptoms were triggered during the full 3-days of testing, the challenge food was removed from the diet and the next challenge food selected to begin a new challenge. If symptoms were triggered on re-introducing a food, the challenge process was stopped to allow symptoms to settle before testing a new food. After the challenges were completed participants could reintroduce FODMAP foods to tolerated levels (as identified through the challenge process) to provide symptom relief in the long term.

At visit 3, 12-weeks after visit 2, final IBS symptom questionnaires and food diaries were collected and personalised dietary advice was provided depending on the outcomes of the food re-introduction process.

FODMAP scores

FODMAP scores were calculated after McIntosh et al. (McIntosh et al. 2017) from the 7 day food diary participants provided at each time point in the study. The FODMAPs were grouped into 7 categories (lactose, fructose, fructans from fruit and vegetables, fructans from cereal, fructans from garlic and onion, galactoligosaccharides and polyols). Depending on the FODMAP type the frequency of ingestion was either scored for daily ingestion (and the score averaged over the week) or for total weekly intake. A low intake was scored 0, moderate intake 1 and a high intake 2. If participants had not provided full information on ingredients on dishes (e.g.ready made meals or in restaurants or take away) the dietitians made assumptions from the typical ingredients included in these dishes. If a food contained a significant amount of more than one FODMAP type it would be scored in both groups. Each food diary was scored between 0-14, the higher the score reflecting a higher intake of wider variety of FODMAPs in the diet. If a participant had a particularly high intake of one particular FODMAP e.g. fructan cereals but a low dietary variety the score could appear lower than a participant with a moderate intake of a variety of the different FODMAP groups.

Median FODMAP scores in 41 subjects undergoing dietary intervention

	Cluster 1	Cluster 2	p
Pre-diet (visit 1)	9<br>Range 5 – 12<br>n = 15	8<br>Range 3 – 13<br>n = 19	0.44
On diet (visit 2)	1<br>Range 0 – 2<br>n = 15	0<br>Range 0 – 6<br>n = 19	0.47
Post diet (visit 3)	7.5<br>Range 6 - 10<br>n = 4	7.5<br>Range 3 – 10<br>n = 12	N.A.

FODMAP scores shown are for subjects with corresponding stool samples at each visit. The p values (Wilcoxon) in the table compare the scores between the clusters at each visit. For each cluster there was a highly significant fall in the score on diet (visit 2) compared to pre-diet (visit 1), p = < 0.00001.

McIntosh K, Reed DE, Schneider T, Dang F, Keshteli AH, De Palma G, et al. FODMAPs alter symptoms and the metabolome of patients with IBS: A randomised controlled trial. Gut. 2017;66(7):1241-51.
